# Microbiomes Associated With the Surfaces of Northern Argentinian Fruits Show a Wide Species Diversity

**DOI:** 10.3389/fmicb.2022.872281

**Published:** 2022-07-11

**Authors:** Louise Vermote, Marko Verce, Fernanda Mozzi, Luc De Vuyst, Stefan Weckx

**Affiliations:** ^1^Faculty of Sciences and Bioengineering Sciences, Research Group of Industrial Microbiology and Food Biotechnology (IMDO), Vrije Universiteit Brussel, Brussels, Belgium; ^2^Technology and Development Laboratory, Centro de Referencia para Lactobacilos (CERELA)-CONICET, San Miguel de Tucumán, Argentina

**Keywords:** fruits, flowers, microbiome, food fermentation, shotgun metagenomics

## Abstract

The fiber, vitamin, and antioxidant contents of fruits contribute to a balanced human diet. In countries such as Argentina, several tropical fruits are witnessing a high yield in the harvest season, with a resulting surplus. Fruit fermentation using autochthonous starter cultures can provide a solution for food waste. However, limited knowledge exists about the microbiota present on the surfaces of fruits and the preceding flowers. In the present exploratory study, the microbiomes associated with the surfaces of tropical fruits from Northern Argentina, such as white guava, passion fruit and papaya were investigated using a shotgun metagenomic sequencing approach. Hereto, one sample composed of 14 white guava fruits, two samples of passion fruits with each two to three fruits representing the almost ripe and ripe stage of maturity, four samples of papaya with each two to three fruits representing the unripe, almost ripe, and ripe stage of maturity were processed, as well as a sample of closed and a sample of open Japanese medlar flowers. A considerable heterogeneity was found in the composition of the fruits’ surface microbiota at the genus and species level. While bacteria dominated the microbiota of the fruits and flowers, a small number of the metagenomic sequence reads corresponded with yeasts and filamentous fungi. A minimal abundance of bacterial species critical in lactic acid and acetic acid fermentations was found. A considerable fraction of the metagenomic sequence reads from the fruits’ surface microbiomes remained unidentified, which suggested that intrinsic species are to be sequenced or discovered.

## Introduction

Food fermentation is a centuries-old biotechnological process initially used to preserve perishable raw materials (Hugenholtz, 2013; Wolfe and Dutton, 2015; Marco et al., 2021). Over the years, more knowledge has been gathered regarding the microorganisms involved in food fermentation processes, being mainly species of lactic acid bacteria (LAB) and yeasts, and in fewer cases also acetic acid bacteria (AAB; Marco et al., 2017, 2021; De Roos and De Vuyst, 2018). The use of autochthonous microbial strains as starter cultures allows to control and even steer such fermentation processes, foremost applied at an industrial scale (Leroy and De Vuyst, 2004). Together with the shelf-life extension resulting from acidification and/or alcoholization and, in some cases, bacteriocin production by the microorganisms involved, fermented foods have additional functional and innovative traits, such as an improved texture and enhanced organoleptic properties as well as natural and artisan characteristics (Leroy and De Vuyst, 2004; Geyzen et al., 2012; Marco et al., 2017, 2021).

Of all food fermentation processes investigated so far, the fermentation of fruits remains largely unexplored. Up to now, focus was on the fermentation of fruit-based products, e.g., smoothies, purees, fresh-cut fruits and fruit juices, inoculated with autochthonous LAB strains isolated from the concomitant fermented fruit-based products (Di Cagno et al., 2010, 2011a,b, 2013, 2016b, 2017). Using culture-dependent enrichment strategies and classical selection on agar media for LAB, the presence of the genera *Apilactobacillus*, *Enterococcus*, *Furfurilactobacillus*, *Lactiplantibacillus*, *Leuconostoc*, *Limosilactobacillus*, *Pediococcus*, and *Weissella* has been shown. However, fruit and fruit juice fermentations not only might deliver a good opportunity to save fruits from low-quality grading and decay due to overproduction, and thus from food losses, but it is also aligned with consumer trends toward the consumption of healthier, fresh-like, high-nutritional-value, ready-to-eat or –drink fermented foods and beverages, including plant-based, non-dairy and non-alcoholic beverages (Di Cagno et al., 2013, 2016a; Randazzo et al., 2016; De Roos and De Vuyst, 2018; Isas et al., 2020; Crespo et al., 2021; Gaglio et al., 2021). Fruit losses occur in particular in countries overproducing fruits, including tropical fruits grown in the Northern part of Argentina, which represent important crops for the region’s agricultural sector (Ruiz Rodríguez et al., 2019). Therefore, it is important to study the taxonomic structure of the microorganisms on the surfaces of such fruits in more detail in view of future controlled fruit (beverage) fermentation processes.

The microbiomes present on fruit surfaces have been extensively studied in the case of fresh fruits or ready-to-eat, minimally processed fruits, mainly to detect pathogenic bacteria and their possible link with human disease outbreaks (Lindow and Brandl, 2003; Delmotte et al., 2009; Leff and Fierer, 2013; Jackson et al., 2015; Wassermann et al., 2019). The microorganisms found have been related to post-harvest processes and actually originate from contacts with contaminated human hands and rinsing water or growth due to non-appropriate transportation and storage conditions (Ailes et al., 2008; Heaton and Jones, 2008; Leff and Fierer, 2013). Previous investigations of the microbiomes present on fruit surfaces also focused on the presence of phytopathogens that cause plant diseases and natural antagonists that could be used as biological control agents for these pathogens (Abdelfattah et al., 2016). Differences in microbiome compositions, comprising bacteria and fungi, between organic and conventionally grown apples and grapes, has been studied as well, using high-throughput, partial 16S rRNA gene (bacteria) and/or (partial) internal transcribed spacer (ITS) region (fungi) sequencing (Ottesen et al., 2009; Abdelfattah et al., 2016; Kecskeméti et al., 2016; Wassermann et al., 2019). These studies have shown distinct microbiomes, which have been related to the fruit parts sampled and, in particular, consist of molds and Gram-negative bacteria. Also, a reduced diversity and evenness for conventionally grown fruits compared with organically grown ones has been shown. As an example, the most abundant fungal genera on the surfaces of grapes are *Alternaria*, *Aureobasidium*, *Botrytis*, and *Cladosporium*, whereas the most abundant bacterial genera are *Erwinia*, *Gluconobacter*, *Massilia*, *Pseudomonas*, and *Sphingomonas* (Kecskeméti et al., 2016). The surfaces of different types of fresh produce have shown a high bacterial diversity with a high relative abundance of *Microbacteriaceae* and *Sphingomonadaceae* on apples and peaches, and of *Bacillaceae* and *Acetobacteraceae* on grapes (Leff and Fierer, 2013). The geographical location of apple cultivation also has an influence on the microbiome with the fungal communities being more affected than the bacterial ones and increasing from country to continental level (Abdelfattah et al., 2021).

Although LAB are assumed to be present on fruit surfaces, which is of interest in view of future fruit fermentation processes, only a limited number of studies has focused on these microbial communities. In general, enumeration of microorganisms on vegetable surfaces has shown that LAB [2.0–4.0 log (CFU/g)] are outnumbered by other aerobic and anaerobic bacteria [5.0–7.0 log (CFU/g)], giving them a serious disadvantage to grow (Di Cagno et al., 2013; Hutkins, 2019). The LAB species identified on common plant tissues in general (e.g., fruits, grains, herbs, and vegetables) and on the surfaces of tropical fruits from Northern Argentina, in particular, belong to the genera *Enterococcus*, *Fructobacillus*, *Lactiplantibacillus*, *Lactobacillus*, *Lactococcus*, *Leuconostoc*, *Levilactobacillus*, and *Weissella* (Ruiz Rodríguez et al., 2019; Yu et al., 2019). Fructophilic LAB species, such as those of the genus *Fructobacillus*, have particularly been isolated through an enrichment strategy from fruits and flowers, reflecting their occurrence in the digestive tract of bees (Endo et al., 2009; Yu et al., 2019).

In addition to the microbiota present on fruit surfaces, also those that are part of the plant phyllosphere, encompassing all aerial parts, have been investigated (Lindow and Brandl, 2003; Whipps et al., 2008; Kim et al., 2012; Vorholt, 2012; Batool et al., 2016; Wassermann et al., 2019). The phyllosphere is colonized by a complex and diverse collection of epiphytic microorganisms, mainly bacteria, as well as filamentous fungi, yeasts, archaea, algae, and viruses. In many cases, the investigation of the phyllosphere has focused on the plant leaves solely (Lindow and Brandl, 2003; Whipps et al., 2008; Delmotte et al., 2009; Kim et al., 2012; Vorholt, 2012). Regarding fungi, yeasts are the major group, whereas molds occur mostly as dormant spores rather than as mycelia (Lindow and Brandl, 2003; Whipps et al., 2008). Flowers, composed of distinct structures that differ in both morphological and physiological properties and of importance for plants because of their role in sexual reproduction, harbor their own specific but different microorganisms that are less studied because of their complexity and transiency (Shade et al., 2013; Aleklett et al., 2014; Junker and Keller, 2015). Genera commonly detected using culture-dependent identification techniques include *Acinetobacter*, *Cryptococcus*, *Metschnikowia*, and *Pseudomonas* (Aleklett et al., 2014). The use of high-throughput, partial 16S rRNA gene sequencing has shown that the phyllosphere has a greater bacterial richness than what has been found previously with culture-dependent methods, although the same highly abundant members have been identified (Lindow and Brandl, 2003; Whipps et al., 2008; Redford et al., 2010; Yashiro et al., 2011; Kim et al., 2012; Jackson et al., 2015). High-throughput, partial 16S rRNA gene sequencing of samples of microbiomes of apple flowers shows a broader bacterial diversity than obtained with culture-dependent methods, and the presence of the understudied *Deinococcus*-*Thermus* and *Saccharibacteria* taxa during temporal sampling (Shade et al., 2013; Aleklett et al., 2014). Flowers that are open for 3 days show a high relative abundance of *Lactobacillus* and *Acetobacter*, which seems to be accompanied with flower decomposition by yeasts (Shade et al., 2013). Furthermore, the fungal diversity is less on open blossoms of apple, pear, and plum than on mature fruits (Vadkertiová et al., 2012). The open flowers harbor *Aureobasidium pullulans* and *Metschnikowia pulcherrima*, whereas *Geotrichum candidum*, *Hanseniaspora guilliermondii*, *Hansenisapora uvarum*, *M. pulcherrima*, *Pichia kluyveri*, *Pichia kudriavzevii* and *Saccharomyces cerevisiae* have been identified on the surfaces and in the flesh of ripe apples, pears, and plums (Vadkertiová et al., 2012).

The aim of the current study was to explore the epiphytic microbial diversity present on the surfaces of wild tropical fruits and flowers harvested in Northern Argentina, using a culture-independent shotgun metagenomic sequencing approach instead of plating or metagenetic analyses. Such an approach will avoid a direct bias imposed by cultivation media used in a culture-dependent (enrichment) strategy (Ruiz Rodríguez et al., 2019) or by the limitations of culture-independent, amplicon-based high-throughput sequencing approaches (Ottesen et al., 2013; Kecskeméti et al., 2016). From an application point of view, species of LAB and AAB detected could eventually be used for the development of a fermented fruit juice as a strategy to cope with fruit surplus during harvest periods, and the knowledge about the microorganisms present can also help in developing strategies to counter post-harvest diseases, possibly resulting in a reduction of food losses (Kusstatscher et al., 2020).

## Materials and Methods

### Fruit and Flower Sampling

White guava (*Psidium guajava*), yellow passion fruit (*Passiflora edulis* var. *flavicarpa*), and papaya (*Carica papaya*), as well as Japanese medlar (loquat) flowers (*Eriobotrya japonica*) were aseptically collected around San Miguel de Tucumán, a city in the Tucumán province in the Northern part of Argentina, known for its particular subtropical climate, in autumn 2014 (white guava), autumn 2015 (Japanese medlar flowers), and autumn 2017 (passion fruit and papaya), resulting in nine samples. The fruits and flowers were picked directly from the trees using sterile gloves, put in sterile stomacher bags, and transported to the laboratory for immediate analysis. For the Japanese medlar flowers, clusters of flowers, both closed and open, were picked, resulting in approximately 10 g of open flowers and approximately 30 g of closed flowers. In the case of white guava, 14 fruit units were combined. For passion fruit, two types of samples, ripe and almost ripe fruits, each containing two to three fruit units, were processed. For papaya, unripe fruits, almost ripe fruits and two samples of ripe fruits, each containing one to two fruit units, were processed. In the laboratory, the fruits and flowers sampled were first washed with 40 mL of a sterile peptone-saline solution [0.1% (m/v) bacteriological peptone (Thermo Fisher Scientific, Waltham, MA, United States) and 0.85% (m/v) sodium chloride (Merck, Darmstadt, Germany)] in a stomacher bag to collect the surface microorganisms. This suspension was then transferred to a centrifuge tube, vortexed for 30 s on the highest setting, and filtered using a 100-μm cell strainer (Avantor, Radnor, PA, United States). Cell pellets were obtained by centrifugation at 6000 *x g* for 20 min at 4°C and washing with 1 mL of TES buffer [0.2 M sucrose (Merck), 1 mM ethylenediaminetetraacetic acid (EDTA; Merck), and 50 mM Tris base (Merck); pH 8.0], after which they were centrifuged again at 6000 *x g* for 20 min at 4°C. The cell pellets thus obtained were stored at –20°C and shipped on dry ice to Belgium for further analysis, without interrupting the cold chain.

### Metagenomic DNA Extraction

Two DNA extraction protocols (tackling both live and intact dead cells) were applied, as samples were taken in three different years and hence processed with adapted protocols. For the white guava and Japanese medlar flowers, a method based on different steps to break the cells, with centrifugation between each step, was used (Vermote et al., 2018), whereas for the passion fruit and papaya samples the same method with an additional chitinase treatment step and with centrifugation only at the end of these steps was applied (De Bruyn et al., 2017; Verce et al., 2021). Briefly, several enzymatic lysis steps (with chitinase, mutanolysin, lysozyme, lyticase, Zymolyase, and proteinase K) were applied, followed by chemical treatment with sodium dodecyl sulphate, and mechanical disruption with glass beads. Then, the DNA was extracted using a phenol:chloroform:isoamyl alcohol treatment. Two DNA purification steps, each with a DNeasy Blood & Tissue Kit (Qiagen, Hilden, Germany), were performed, according to the manufacturer’s instructions, with in between an in-solution RNase treatment (Thermo Fischer Scientific, Waltham, MA, United States). The quality of the DNA was visually assessed by agarose gel electrophoresis. The DNA purity was measured with a NanoDrop 2000 spectrophotometer and the DNA concentration was measured with a Qubit 2.0 fluorometer using a Qubit dsDNA HS Assay Kit (all from Thermo Fisher Scientific).

### Shotgun Metagenomic Sequencing

DNA fragment libraries of approximately 350 bp were made, as described previously (Vermote et al., 2018). All reagents, kits, and equipment described below were purchased from Thermo Fischer Scientific (Waltham, MA, United States), unless stated otherwise. Sequencing was performed using an Ion Torrent Personal Genome Machine (PGM) with a HiQ (white guava and medlar flowers) or HiQ View (papaya and passion fruit) sequencing kit. For each sample sequenced, one chip, an Ion 316 (white guava), an Ion 316 v2 (medlar flowers), or an Ion 316 v2 BC (passion fruit and papaya), was used, as the samples were processed in three different years. All steps were performed following the manufacturer’s instructions.

### Bioinformatic Analysis

#### Sequence Reads Quality Trimming

The quality of the metagenomic sequence reads obtained was assessed using FastQC (version 0.10.1; Andrews, 2010). Based on these results, the metagenomic sequence reads were trimmed using prinseq-lite (version 0.20.2; Schmieder and Edwards, 2011), using a sliding window of 10 bases with steps of 4 bases and a minimum average quality score of 20. Further, the minimum quality score at both ends of the metagenomic sequence reads was set to 20 and the minimum length of the resulting trimmed sequences to 50 bases. The quality-trimmed metagenomic sequence reads are further referred to as MSRs.

#### Taxonomic Classification on Genus Level Based on All Metagenomic Sequence Reads

The MSR data sets were used for taxonomic classification on genus level, using four different methods, to obtain algorithm- and database-independent results (Illeghems et al., 2012; Verce et al., 2019). Two methods were based on nucleotide-nucleotide classification and two were based on nucleotide-protein classification. For the first nucleotide-nucleotide classification method, the MSRs were aligned to the non-redundant nucleotide (nt) database of the National Center for Biotechnology Information (NCBI; Bethesda, MD, United States), using blastn (Altschul et al., 1990). The output was further analyzed using MEGAN (version 6.7.11; Huson et al., 2007), with the minimum score set to 100. Based on these results, a rarefaction analysis was performed for each data set to check whether sufficient MSRs were obtained. The second nucleotide-nucleotide classification method used was Kraken (version 0.10.5-beta; Wood and Salzberg, 2014), using a custom-made database consisting of bacterial, archaeal, and lower eukaryotic genome assemblies from type material from the NCBI assembly database. The first nucleotide-protein classification method used was DIAMOND (version 0.9.22; Buchfink et al., 2015), aligning the MSRs to the non-redundant protein (nr) database of the NCBI. The output was further analyzed using MEGAN, with the same parameters as mentioned above. The second nucleotide-protein classification method used was Kaiju (version 1.5.0; Menzel et al., 2016), using a customized database consisting of bacterial, archaeal, and lower eukaryotic protein sequences obtained from the NCBI nr database. The output of all classification methods was analyzed with R (R Core Team, 2020) in RStudio (RStudio Team., 2020), using the packages reshape (Wickham, 2007), tidyverse (Wickham et al., 2019), and lazyeval (Wickham, 2019).

#### Removal of Plant-Derived Metagenomic Sequence Reads

To allow for an efficient computation of the sequence alignments, of which the output was to be used as input for fragment recruitment plotting, the MSRs were first aligned to the genome sequences of the plants corresponding with the samples, or in the case a genome sequence was not available to the genome sequence of a closely related plant species, to identify and remove those plant-assigned reads from the data sets. The MSRs aligning with a minimum percentage identity of 60% and a minimum query coverage of 60% were removed. For each fruit sample, the corresponding plant’s genome sequence was obtained from the NCBI genome database. For white guava, the *Ps. guajava* Zhenzhu genome (accession number GCA_002914565.1), for passion fruit, the *Pa. edulis* CGPA1 genome (accession number GCA_002156105.1), and for papaya, the *C. papaya* SunUp genome (accession number GCA_000150535.1), were used. As there was no genome sequence available for Japanese medlar at the time of analysis, the genomes of the two closest related species were used, namely *Malus domestica* Golden Delicious (accession number GCA_00148765.2) and *Pyrus bretschneideri* Dangshansuli (accession number GCF_000315295.1).

#### Taxonomic Classification on Genus and Species Level Using Fragment Recruitment Plots

The plant sequence-depleted MSR data sets were subsequently aligned, using blastn, to a custom-made database, containing a representative genome sequence (if available) of each species of the genera detected in at least one of the samples with at least one of the four classification methods mentioned above and with a relative abundance of at least 0.1% (Verce et al., 2019). The resulting custom-made database was extended with a representative genome sequence (if available) of species of the genera belonging to the *Acetobacteraceae* family and genera belonging to the LAB group. The blastn output was filtered in such a way that for each MSR only the best hit with a minimum sequence identity of 60% and a minimum query coverage of 60% was considered. For each genome, the average nucleotide identity (ANI) of the aligned MSRs was calculated and the actual fragment recruitment plots (FRPs) were plotted using R (version 4.0.3; R Core Team, 2020), RStudio (version 1.3.1093; RStudio Team., 2020), and the R packages scales (version 1.1.1; Wickham and Seidel, 2020), tidyverse (version 1.3.1; Wickham et al., 2019), and gridExtra (version 2.3; Auguie, 2017), whereby each dot in the plot represented one MSR. To obtain a quantitative measure to decide whether or not a species recruited sufficient reads to consider it present, each genome sequence was divided into ten sections, and the distribution of the reads in the different sections (RSD) was calculated by dividing the standard deviation of the number of reads per section by the mean of the number of reads per section. The presence of a species in a sample was manually decided based on its ANI score, an RSD value of less than 0.8, and a minimum number of MSRs aligning to a genome sequence of 1000. In addition, MSRs were only considered if they had a minimum sequence identity of 60%.

### Statistical Analysis

Principal component analysis (PCA) was performed on the plant sequence-depleted MSR dataset obtained from fragment recruitment plotting on genus level, taking into account only the genera that were detected with a relative abundance of at least 0.9% in at least one of the samples, to identify patterns associated with the fruits and flowers sampled. Intra-sample diversity (alpha-diversity) was assessed by calculating the Simpson (diversity) and Pielou (evenness) indexes (Mouillot and Leprêtre, 1999). Inter-sample diversity (beta-diversity) was assessed to determine the differences between all samples by permutational multivariate analysis of variance (PERMANOVA), based on Bray-Curtis dissimilarity scores (Anderson, 2017). Doing so, it was taken into account that the samples of the same fruit and flower types were collected in the same year, enabling within-species comparison, whereas samples of the different fruits and flower types were collected in different years. Subsequently, series of pairwise PERMANOVA comparisons and a similarity percentage analysis (SIMPER; Clarke, 1993) was performed. A significance level of 0.05 was considered for all statistical procedures. The PCA plot and statistical analyses performed were executed using the ggplot2 (version 3.3.5; Wickham, 2016), ggrepel (version 0.9.1; Slowikowski, 2020), vegan (version 2.5.7; Oksanen et al., 2019), and RVAideMemoire (version 0.9.81; Hervé, 2021) packages in RStudio.

## Results

### Sampling, Metagenomic DNA Extraction, and Shotgun Metagenomic Sequencing

White guava, yellow passion fruit, papaya, and Japanese medlar flowers were collected during several field experiments around San Miguel de Tucumán, Northern Argentina, in the period 2013–2017, which resulted in nine samples. After cell pellet collection, metagenomic DNA extraction, and shotgun metagenomic sequencing using an Ion Torrent PGM platform, nine MSR data sets were obtained, with the number of high-quality reads ranging between 2.5 and 4.0 million, representing between 477 and 971 Mb of sequence data (Table 1). Rarefaction analysis showed that a sufficient amount of MSRs were obtained (data not shown).

**TABLE 1:** Number of metagenomic sequence reads (MSRs) and total number of bases obtained for various fruit and flower samples after shotgun metagenomic sequencing with an Ion Torrent PGM platform, as well as the total number of MSRs and number of bases obtained after quality trimming, and number of MSRs aligned to plant genomes.

Sample	Total number of MSRs	Total number of bases (Mb)	Total number of MSRs after trimming	Total number of bases (Mb) after trimming	Number of MSRs aligned to plant genomes
WGF	2,835,153	643	2,530,763	477	30,743
MFC	3,238,565	847	3,028,844	716	74,827
MFO	3,212,647	932	3,056,370	816	374,259
PFA	3,840,122	1130	3,764,906	964	11,559
PFR	3,512,139	1000	3,400,841	860	268,483
PAU	4,074,414	1048	4,003,147	922	958,779
PAA	3,749,566	987	3,664,582	880	634,758
PAR1	2,977,156	639	2,782,991	533	304,202
PAR2	4,082,505	1154	4,012,239	971	49,089

*WGF, white guava fruits; MFC, closed medlar flowers; MFO, open medlar flowers; PFA, almost ripe passion fruits; PFR, ripe passion fruits; PAU, unripe papaya fruits; PAA, almost ripe papaya fruits; PAR1 and PAR2, ripe papaya fruits.*

### Taxonomic Classification

To assess the nature of the microorganisms present on the surfaces of the fruits and flowers sampled, the nine MSR data sets obtained were analyzed using four different methods for taxonomic classification at genus level. The use of various algorithms and databases enabled to obtain results with as little biases as possible. The results obtained for the genus-level taxonomic classification were subsequently used for a more detailed taxonomic classification at species level using FRPs.

#### Genus-Level Taxonomic Classification

**FIGURE 1:**
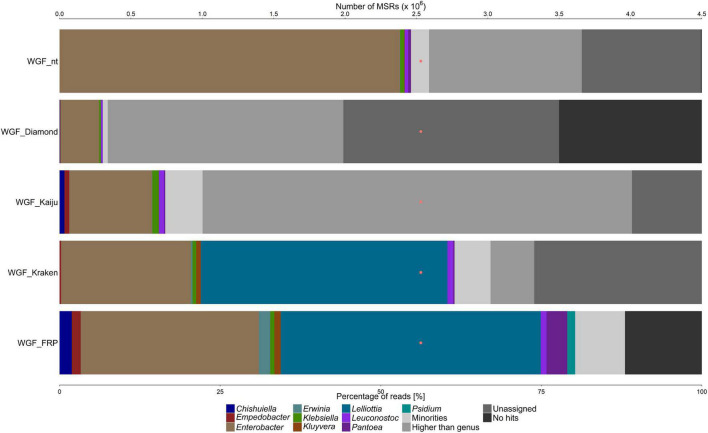
Taxonomic classification of the microbiota on white guava (WGF sample), based on metagenomic sequence reads (MSRs), using a sequence alignment approach based on blastn (with the nt database) and DIAMOND (with the nr database), Kaiju and Kraken (with a customized database containing bacterial, archaeal, and lower eukaryotic sequences), and fragment recruitment plotting (FRP). The category “Minorities” represents all genera present with a relative abundance below 0.9% for all methods used. The category “Higher than genus” represents all assigned taxonomic levels above genus level. The category “Unassigned” represents reads that were not assigned to any taxonomic level. The category “No hits” includes all reads that could not be classified at all. The orange dots represent the number of MSRs that were used for the taxonomic classification.

**FIGURE 2:**
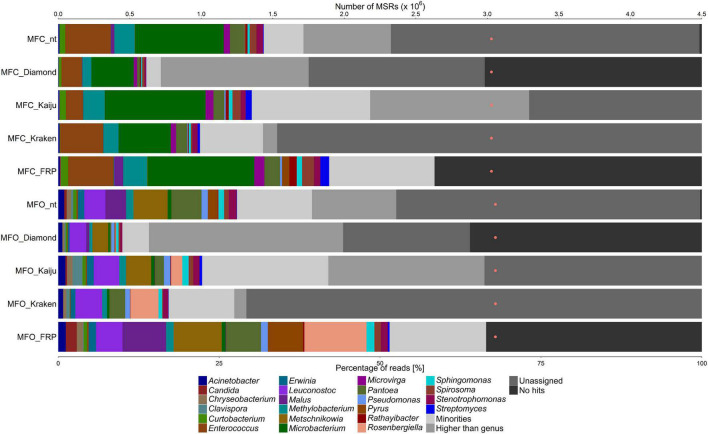
Taxonomic classification of the microbiota on Japanese closed and open medlar flowers (MFC and MFO samples, respectively), based on metagenomic sequence reads (MSRs), using a sequence alignment approach based on blastn (with the nt database) and DIAMOND (with the nr database), Kaiju and Kraken (with a customized database containing bacterial, archaeal, and lower eukaryotic sequences), and fragment recruitment plotting (FRP). The category “Minorities” represents all genera present with a relative abundance below 0.9% for all methods used. The category “Higher than genus” represents all assigned taxonomic levels above genus level. The category “Unassigned” represents reads that were not assigned to any taxonomic level. The category “No hits” includes all reads that could not be classified at all. The orange dots represent the number of MSRs that were used for the taxonomic classification.

**FIGURE 3:**
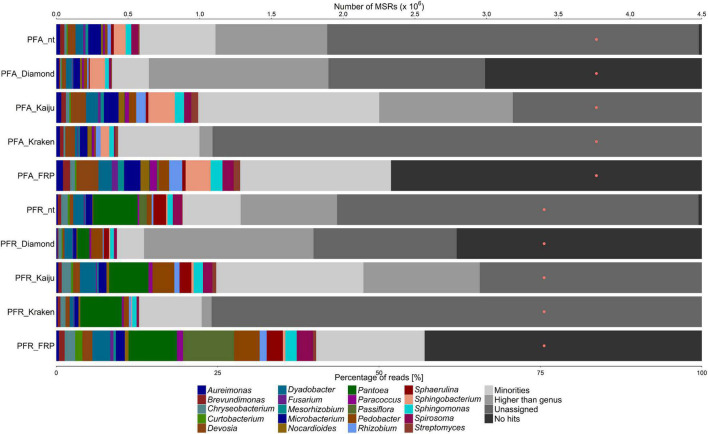
Taxonomic classification of the microbiota on almost ripe and ripe passion fruits (PFA and PFR samples, respectively), based on metagenomic sequence reads (MSRs), using a sequence alignment approach based on blastn (with the nt database) and DIAMOND (with the nr database), Kaiju and Kraken (with a customized database containing bacterial, archaeal, and lower eukaryotic sequences), and fragment recruitment plotting (FRP). The category “Minorities” represents all genera present with a relative abundance below 0.9% for all methods used. The category “Higher than genus” represents all assigned taxonomic levels above genus level. The category “Unassigned” represents reads that were not assigned to any taxonomic level. The category “No hits” includes all reads that could not be classified at all. The orange dots represent the number of MSRs that were used for the taxonomic classification.

**FIGURE 4:**
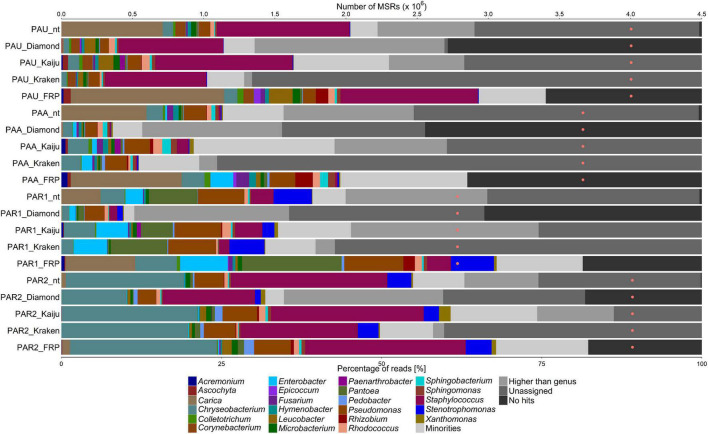
Taxonomic classification of the microbiota on unripe, almost ripe, and ripe papaya (PAU, PAA, and PAR1/PAR2 samples), based on metagenomic sequence reads (MSRs), using a sequence alignment approach blastn (with the nt database) and DIAMOND (with the nr database), Kaiju and Kraken (with a customized database of bacterial, archaeal, and lower eukaryotic sequences), and fragment recruitment plotting (FRP). The category “Minorities” represents all genera present with a relative abundance below 0.9% for all methods used. The category “Higher than genus” represents all assigned taxonomic levels above genus level. The category “Unassigned” represents reads that were not assigned to any taxonomic level. The category “No hits” includes all reads that could not be classified at all. The orange dots represent the number of MSRs that were used for the taxonomic classification.

All fruit and flower samples showed a high diversity of microorganisms, except for the white guava fruit sample (WGF) that had a low one (Figures 1–4). Further, all samples had a high proportion of MSRs (25–50% of the total number) that gave no sequence alignment hit or could not be assigned at genus level.

For the WGF sample, MSRs were assigned to *Enterobacter* and *Klebsiella*, and a small number of reads to *Leuconostoc*, with all methods used (Figure 1). MSRs were also assigned to *Pantoea* with all methods used, except for DIAMOND. MSRs were assigned to *Lelliottia* and *Kluyvera* with Kraken only and to *Chishuiella* and *Empedobacter* with Kaiju only.

The taxonomic analysis of the MSRs obtained from the Japanese medlar flower samples showed differences in the genera present between closed (MFC) and open (MFO) flowers (Figure 2). For the MFC sample, MSRs were assigned to, in decreasing order of relative abundance, *Microbacterium*, *Enterococcus*, *Methylobacterium*, *Pantoea*, *Stenotrophomonas*, *Curtobacterium*, *Microvirga*, *Sphingomonas*, and *Spirosoma*, with all methods used. *Acinetobacter*, *Rathayibacter*, and *Streptomyces* were detected in lower relative abundances, but not with all methods used. For the MFO sample, MSRs were assigned to, in decreasing order of relative abundance, *Leuconostoc*, *Pantoea*, *Stenotrophomonas*, *Methylobacterium*, *Pseudomonas*, *Acinetobacter*, *Erwinia*, *Sphingomonas*, *Chryseobacterium*, *Curtobacterium*, and *Spirosoma*, with all methods used. *Metschnikowia* was detected with all methods, except for Kraken, and *Rosenbergiella* was detected with Kaiju and Kraken only.

For both passion fruit samples, i.e., almost ripe (PFA) and ripe (PFR) passion fruits, the microbial communities found displayed a high but similar diversity (Figure 3). *Aureimonas*, *Brevundimonas*, *Chryseobacterium*, *Devosia*, *Dyadobacter*, *Microbacterium*, *Nocardioides*, *Pedobacter*, *Rhizobium*, *Sphingomonas*, and *Spirosoma* were found in both samples, with all methods used, whereas *Sphaerulina* was detected in both samples with all methods used, except for Kraken. *Mesorhizobium* was found in both samples with all methods used, except for DIAMOND (both samples) and Kraken (PFR sample). *Fusarium* was detected in both samples with the blastn-based method and Kaiju, whereas *Streptomyces* was found in both samples with Kaiju only. *Sphingobacterium* was only found in the PFA sample, with all methods used, and *Pantoea* was only found in the PFR sample, with all methods used.

For the set of the papaya samples, comprising samples from unripe (PAU), almost ripe (PAA), and ripe (PAR1, PAR2) fruits, *Chryseobacterium*, *Microbacterium*, *Pantoea*, *Pseudomonas*, *Rhodococcus*, *Sphingomonas*, *Staphylococcus*, and *Xanthomonas* were found, with varying relative abundances in all samples, with all methods used (Figure 4). *Pedobacter* was found in the PAA and PAR1 samples with Kaiju and Kraken, and in the PAR2 sample with all methods used. *Sphingobacterium* was found in all samples, with all methods, except for the PAR1 sample when using Kraken. *Leucobacter* was found in all samples, with all methods used, except for the blastn-based method. For the PAA and PAR1 samples, the relative abundances found with DIAMOND and Kraken were lower. *Enterobacter* was found in the PAA and PAR1 samples, with all methods used, and in lower relative abundances in all other samples. *Stenotrophomonas* was found in the PAR1 and PAR2 samples and in lower relative abundances in the PAU and PAA samples, with all methods used, except for DIAMOND. *Acremonium* and *Ascochyta* were found with Kaiju in the PAU, PAA, and PAR1 samples. *Ascochyta* was also found with Kaiju in the PAR2 sample. *Colletotrichum* was found, with all methods used, in the PAU, PAA, and PAR1 samples. *Corynebacterium* was found, with all methods used, except for Kraken, in the PAU sample. *Fusarium* was found in the PAU and PAA samples, with all methods used, and with the blastn-based method and Kaiju in the PAR1 sample. *Hymenobacter* was found in the PAU and PAA samples, with all methods used, and in the PAR1 sample with the blastn-based method and Kaiju. *Paenarthrobacter* was found in all samples with Kaiju only. *Rhizobium* was found in all samples, except for the PAU sample, with Kaiju and Kraken. *Xanthomonas* was found in the PAA, PAR1, and PAR2 samples, with all methods used, except for Kraken for the PAA and PAR1 samples and DIAMOND for the PAR1 sample.

#### Removal of Plant-Derived Metagenomic Sequence Reads

Before the MSRs were used for a detailed taxonomic classification using fragment recruitment plots, plant-related sequences needed to be removed. Hereto, the MSR data sets were aligned to concomitant fruit genome sequences using blastn (Table 1). For the WGF sample, 1.37% of all MSRs were assigned to *Psidium*, the genus to which white guava belongs (Figure 1). For the MFC sample, 1.34% of all MSRs were assigned to *Malus* and 1.12% to *Pyrus*, and for the MFO sample, 6.77% of all MSRs were assigned to *Malus* and 5.47% to *Pyrus* (Figure 2). For the passion fruit samples, the PFA and PFR samples contained 0.31 and 7.89% of all MSRs assigned to *Passiflora* (Figure 3). For the papaya samples, the PAU sample contained 14.89% of all MSRs, the PAA sample 10.18% of all MSRs, the PAR1 sample 6.81% of all MSRs, and the PAR2 sample 0.75% of all MSRs that aligned to *Carica* (Figure 4).

#### Genus- and Species-Level Taxonomic Classification Using Fragment Recruitment Plots

Aligning the plant sequence-depleted MSR data sets to a custom-made genome sequence database, which was constructed based on the data of the genus-level taxonomic classification, resulted in an output that was used to make FRPs. In general, the taxonomic classification at genus level using FRPs resulted in a higher percentage of assigned reads for all samples compared with the four methods used for genus-level classification, ranging from 51.85 to 88.02% of all MSRs (Figures 1–4). Overall, the genera detected with the four classification methods mentioned above were also detected by means of the FRPs, and this with similar or even higher relative abundances. The use of one comprehensive database to generate the FRPs for all samples resulted in the detection of additional genera per sample, such as *Erwinia* in the WGF sample. All resulting FRPs were visually inspected and the species with an ANI value above 90% are reported in Tables 2, 3. The number of species detected and the total percentage of MSRs these species accounted for differed between the samples, ranging from only 4.47% (23 species) in the case of the PFA sample to 68.19% (17 species) in the case of the WGF sample. For the PFA sample, most MSRs that aligned at species level with an ANI value lower than 90% were not further considered. For most samples, most species found with an ANI value of more than 90% belonged to the γ-proteobacteria (e.g., *Enterobacter*, *Pantoea*, *Pseudomonas*, and *Stenotrophomonas*). In some cases, two clouds of dots were obtained in the FRPs, typically one at an overall high percentage sequence identity and one at an overall lower percentage sequence identity. The former cloud represented MSRs that most likely came from a species that was present in the sample and whose genome was present in the database used for fragment recruitment plotting. Though, these MSRs could also come from a species that was phylogenetically closely related with that species but for which the genome sequence was not yet available. The latter cloud represented MSRs that came from a species that was phylogenetically more distant from the species whose genome sequence the MSRs aligned to. This could point to an underrepresentation of appropriate species in the genome sequence databases. Overall, the results of the FRPs showed that there was not only a large species diversity per sample but also between samples of the same fruit or flower type (Tables 2, 3 and Figures 1–4).

**TABLE 2:** Bacterial species found through shotgun metagenomic sequencing, using a fragment recruitment plotting approach, and sorted by class.

Taxonomy	WGF	MFC	MFO	PFA	PFR	PAU	PAA	PAR1	PAR2
**Actinobacteria**	**0.10**	**1.19**	**0.44**	**1.39**	**1.54**	**1.45**	**0.32**	**0.57**	**1.11**
*Actinosynnema mirum*									0.40
*Aeromicrobium massiliense*				0.26	0.20				
*Agrococcus jejuensis*					0.12				
*Brachybacterium paraconglomeratum*				0.11			0.03		0.06
*Cellulosimicrobium cellulans*			0.06						
*Corynebacterium casei*						1.24			
*Corynebacterium glyciniphilum*						0.13			
*Curtobacterium citreum*					0.05				
*Curtobacterium flaccumfaciens*	0.06				0.04				
*Curtobacterium luteum*					0.03				
*Curtobacterium oceanosedimentum*			0.09	0.09	0.18				
*Curtobacterium plantarum*		0.04			0.77		0.03	0.18	0.04
*Curtobacterium pusillum*		0.33	0.18						
*Friedmanniella sagamiharensis*	0.04								
*Gordonia lacunae*								0.15	
*Gordonia terrae*				0.16	0.03		0.10	0.24*	0.27*
*Leucobacter japonicus*			0.06						
*Leucobacter musarum*			0.05						
*Microbacterium lemovicicum*		0.82							
*Microbacterium oxydans*				0.34	0.12				
*Nocardioides alkalitolerans*				0.15*					
*Pseudonocardia alni*				0.11			0.16		0.31
*Rhodococcus kroppenstedtii*									0.03
*Sanguibacter keddieii*				0.11		0.08			
*Streptomyces cyaneofuscatus*				0.06					
**Bacteroidetes**	**0.00**	**0.00**	**0.28**	**1.66**	**0.81**	**0.21**	**3.37**	**0.00**	**17.55**
*Chryseobacerium artocarpi*							1.27		15.59
*Chryseobacterium balustinum*							0.12		
*Chryseobacterium cucumeris*				0.03					
*Chryseobacterium halperniae*			0.09						
*Chryseobacterium indoltheticum*					0.11*		0.48		
*Chryseobacterium piscium*							0.13		
*Chryseobacterium scophthalmum*			0.07				0.76		
*Chryseobacterium ureilyticum*							0.15		1.77
*Pedobacter agri*				0.17*	0.60*		0.04		
*Sphingobacterium deserti*				1.46*	0.10	0.21	0.42*		0.13
*Sphingobacterium siyangense*			0.12						
*Spirosoma rigui*									0.06*
**Deinococcus-Thermus**	**0.00**	**0.00**	**0.00**	**0.00**	**0.00**	**0.08**	**0.22**	**0.00**	**0.00**
*Deinococcus gobiensis*						0.08	0.22		
**Firmicutes**	**0.73**	**6.18**	**4.28**	**0.13**	**1.32**	**19.48**	**0.83**	**1.46**	**23.21**
*Enterococcus casseliflavus*			0.10		0.03		0.08		0.04
*Enterococcus faecium*		6.18							
*Exiguobacterium acetylicum*					0.05		0.05		
*Exiguobacterium enclense*					0.03		0.04		
*Exiguobacterium indicum*					0.03		0.05		
*Lactococcus lactis* subsp. *hordniae*			0.05				0.03		0.05
*Lactococcus lactis* subsp. *lactis*			0.14				0.06	0.04	0.14
*Leuconostoc citreum*								0.05	
*Leuconostoc pseudomesenteroides*	0.73		3.38				0.06		0.05
*Mammaliicoccus sciuri*					0.05	13.81	0.15	0.93	15.72
*Saccharibacillus sacchari*					0.16				
*Staphylococcus gallinarum*				0.03					
*Staphylococcus pragensis*				0.03					
*Staphylococcus schleiferi*					0.93		0.21	0.91	
*Staphylococcus xylosus*				0.07		5.67	0.10*	2.32	7.21*
*Weissella bombi*			0.03						
*Weissella cibaria*			0.58		0.04				
**Flavobacteria**	**0.00**	**0.00**	**0.00**	**0.24**	**0.00**	**0.00**	**0.00**	**0.00**	**0.00**
*Dyadobacter fermentans*				0.24*					
**Alphaproteobacteria**	**0.15**	**0.20**	**0.12**	**0.73**	**0.33**	**0.23**	**0.38**	**0.04**	**0.25**
*Asaia platycodi*		0.13	0.04						
*Aureimonas altamirensis*				0.36*	0.12		0.09		0.04
*Aureimonas ferruginea*				0.17*	0.07				0.07
*Aureimonas ureilytica*				0.20*					
*Brevundimonas vesicularis*							0.03	0.04	
*Gluconobacter frateurii*	0.08		0.08						
*Gluconobacter kondonii*	0.07								
*Ochrobactrum pseudogrignonense*		0.04					0.15		0.09
*Sphingomonas aeria*					0.14	0.07	0.11		0.05
*Sphingomonas paucimobilis*		0.03							
*Sphingomonas rubra*						0.16*			
**Betaproteobacteria**	**0.00**	**0.00**	**0.00**	**0.00**	**0.00**	**0.00**	**0.03**	**0.10**	**0.29**
*Achromobacter mucicolens*									0.08
*Acidovorax avenae*								0.05	0.10
*Acidovorax citrulli*								0.05	0.11
*Massilia aurea*							0.03		
**Gammaproteobacteria**	**67.07**	**2.62**	**4.43**	**0.06**	**5.97**	**1.91**	**6.89**	**27.74**	**9.26**
*Acinetobacter bereziniae*			0.22						
*Acinetobacter johnsonii*		0.13	0.05						
*Acinetobacter radioresistens*		0.04							
*Acinetobacter soli*			0.27						
*Enterobacter asburiae*	1.80							0.09	
*Enterobacter bugandensis*	1.18							0.32	
*Enterobacter cancerogenus*			0.04					2.45	
*Enterobacter chengduensis*	2.09								
*Enterobacter chuandaensis*	0.99							0.04	
*Enterobacter hormaechei*							0.60		
*Enterobacter huaxiensis*			0.24	0.06				0.07	
*Enterobacter kobei*	0.47								
*Enterobacter ludwigii*			0.04						
*Enterobacter mori*								1.44	
*Enterobacter roggenkampii*	16.47							0.05	
*Enterobacter sichuanensis*	1.00								0.03
*Enterobacter tabaci*								2.59	
*Enterobacter xiangfangensis*							2.47	0.07	
*Erwinia dacicola*			0.18						
*Erwinia iniecta*			0.28						
*Kluyvera ascorbata*			0.04						
*Kluyvera cryocrescens*			0.04						
*Kosakonia cowanii*			0.10			0.10	0.56	0.15	0.57
*Leclercia adecarboxylata*									0.05
*Lelliottia nimipressularisa*	40.42								
*Pantoea agglomerans*					0.74		0.03	0.14	0.04
*Pantoea ananatis*					2.40			0.12	
*Pantoea anthophila*			0.14		0.20			2.12	
*Pantoea coffeiphila*			1.66						
*Pantoea deleyi*					0.09				
*Pantoea dispersa*	0.04								0.03
*Pantoea eucrina*					0.18	0.08			
*Pantoea sesami*	2.56								
*Pantoea stewartii* subsp. *indologenes*					0.05				
*Pantoea vagans*		1.70	0.14		1.85	0.20	0.23	2.23	0.18
*Pantoea wallisii*								2.68*	0.56
*Pseudomonas argentinensis*						0.52	1.20	0.91	0.46
*Pseudomonas coleopterorum*			0.05						
*Pseudomonas entomophila*								0.15	
*Pseudomonas extremorientalis*								0.24	
*Pseudomonas fulva*			0.07				0.19	0.17	2.39
*Pseudomonas helleri*							0.05	1.49	
*Pseudomonas oleovorans*			0.05			0.12	0.06	0.19	0.07
*Pseudomonas oryzihabitans*			0.06			0.13	0.07	0.19	0.07
*Pseudomonas parafulva*			0.06						
*Pseudomonas psychrotolerans*			0.06			0.14	0.06	0.20	0.07
*Pseudomonas punonensis*						0.17	0.35	0.30	0.14
*Pseudomonas putida*								1.16*	
*Pseudomonas soli*								0.20	0.19
*Pseudomonas straminea*						0.45	0.96	0.78	0.4
*Serratia liquefaciens*	0.05								0.06
*Serratia marcescens*								0.06	
*Serratia nematophilia*								0.05	
*Serratia ureilytica*								0.41	0.05
*Stenotrophomonas bentonitica*		0.04	0.14				0.06	0.21	
*Stenotrophomonas indicatrix*								0.26	0.29
*Stenotrophomonas lactitubi*								2.52	0.45
*Stenotrophomonas maltophilia*		0.50	0.25					3.47	1.66
*Stenotrophomonas pavanii*		0.21	0.22						1.19
*Stenotrophomonas rhizophila*					0.46				
*Xanthomonas arboricola*			0.03						
*Xanthomonas retroflexus*								0.22	0.31

*Only species with an average percentage sequence identity of more than 90% or species for which one of the read clouds had a sequence identity of more than 90% were considered. A dark green cell denotes an average percentage sequence identity between 95 and 100%; a light green cell denotes an average percentage sequence identity between 90 and 95%; and a yellow cell denotes an average percentage sequence identity between 80 and 90%. An asterisk denotes the presence of two read clouds in the fragment recruitment plot. The numbers in the cells correspond to the relative percentage of reads aligning to a species. For each class, the total relative percentage is reported. WGF, white guava fruits; MFC, closed medlar flowers; MFO, open medlar flowers; PFA, almost ripe passion fruits; PFR, ripe passion fruits; PAU, unripe papaya fruits; PAA, almost ripe papaya fruits; PAR1 and PAR2, ripe papaya fruits.*

**TABLE 3:** Fungal species found through shotgun metagenomic sequencing, using a fragment recruitment plotting approach, and sorted by phylum.

Taxonomy	WGF	MFC	MFO	PFA	PFR	PAU	PAA	PAR1	PAR2
**Ascomycota**	**0.14**	**0.05**	**3.69**	**0.26**	**0.30**	**0.28**	**2.25**	**0.14**	**0.39**
*Alternaria alternata*				0.05	0.05	0.06	0.11		
*Alternaria arborescens*							0.05		
*Alternaria tenuissima*				0.04	0.04	0.06	0.11		
*Candida hawaiiana*			0.16						
*Colletotrichum nymphaeae*			0.07						
*Debaryomyces maramus*		0.05							
*Epicoccum nigrum*							0.28*		
*Fusarium equiseti*					0.07		0.70		
*Fusarium incarnatum*	0.14		0.14	0.17	0.10	0.16	0.27		
*Hanseniaspora opuntiae*							0.64	0.14	0.25
*Hanseniaspora uvarum*			0.47		0.04		0.09		0.14
*Metschnikowia kipukae*			0.23*						
*Metschnikowia reukaufii*			2.62*						
**Basidiomycota**	**0.00**	**0.00**	**0.33**	**0.00**	**0.00**	**0.09**	**0.65**	**0.13**	**0.00**
*Moesziomyces aphidis*						0.09*	0.10*	0.13*	
*Pseudozyma hubeiensis*			0.33				0.55*		

*Only species with an average percentage sequence identity of more than 90% or species for which one of the read clouds had a sequence identity of more than 90% were considered. A dark green cell denotes an average percentage sequence identity between 95 and 100%; a light green cell denotes an average percentage sequence identity between 90 and 95%; a yellow cell denotes an average percentage sequence identity between 80 and 90%; and an orange cell denotes an average percentage sequence identity between 70 and 80%. An asterisk denotes the presence of two read clouds in the fragment recruitment plot. The numbers in the cells correspond to the relative percentage of reads aligning to a species. For each phylum, the total relative percentage is reported. WGF, white guava fruits; MFC, closed medlar flowers; MFO, open medlar flowers; PFA, almost ripe passion fruits; PFR, ripe passion fruits; PAU, unripe papaya fruits; PAA, almost ripe papaya fruits; PAR1 and PAR2, ripe papaya fruits.*

### Statistical Processing of the Data

**FIGURE 5:**
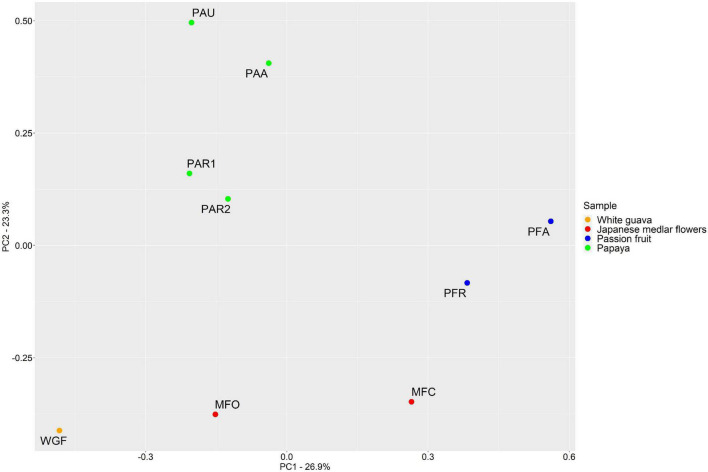
Principal component analysis (PCA) of the microbial composition of various fruits and flowers. The plot is based on the relative abundances of all species, detected by fragment recruitment plotting after shotgun metagenomic sequencing. Samples are color-coded according to the sample type. WGF, white guava fruits; MFC, closed medlar flowers; MFO, open medlar flowers; PFA, almost ripe passion fruits; PFR, ripe passion fruits; PAU, unripe papaya fruits; PAA, almost ripe papaya fruits; PAR1 and PAR2, ripe papaya fruits.

Two principal components (PCs) were obtained after a PCA of the relative abundances of the genera found by means of FRPs, which covered 50.2% of the total variance (Figure 5). PC1 was characterized by a negative loading for *Erwinia* and positive loadings, in decreasing order of contribution, for *Rhodococcus*, *Colletotrichum*, *Rhizobium*, *Hymenobacter*, *Epicoccum*, *Ascochyta*, *Acremonium*, *Leucobacter*, and *Fusarium*. PC2 was characterized by positive loadings for *Aureimonas*, *Nocardioides*, *Mesorhizobium*, *Spirosoma*, *Sphingomonas*, *Devosia*, *Paracoccus*, *Brevundimonas*, *Dyadobacter*, and *Streptomyces*. The samples were well separated according to the concomitant fruit species. The samples from papaya were more associated with the negative values of PC1 and the positive values of PC2, whereas the ones from the white guava fruits were more associated with the negative values of PC1 and the negative values of PC2. The samples from the passion fruits were more associated with the positive values of PC1, whereas those from the Japanese medlar flowers were more associated with the negative values of PC2.

Intra-sample diversity showed that the WGF sample had the lowest genus diversity and evenness compared to the other samples analyzed (Table 4). The genus diversity was the highest for the PFA and PAA samples, whereas their evenness was similar. The PAR1 and PAR2 samples showed differences in both genus diversity and evenness, whereby the values for the PAR2 sample were lower than those for the PAR1 sample. The diversity of the PAR2 sample was comparable to the one of the unripe papaya sample (PAU). Microbial inter-species diversity (PERMANOVA) showed a significant difference (*p* < 0.05) between the different samples based on the concomitant fruit species, with the WGF sample being different from the other fruit samples. SIMPER highlighted that differences in microbial communities at genus level were primarily ascribed to the presence of *Lelliottia* in the white guava sample; the presence of, in decreasing order of contribution, *Staphylococcus*, *Chryseobacterium*, *Pantoea*, and *Pseudomonas* in the papaya samples; the presence of *Dyadobacter* in the passion fruit samples, and the presence of *Microbacterium*, *Rosenbergiella*, *Metschnikowia*, and *Enterococcus* in the Japanese medlar flower samples.

**TABLE 4:** Alpha diversity metrics based on the relative abundances of genera, detected using fragment recruitment plotting, in the different fruit samples examined through shotgun metagenomic sequencing.

Sample	Simpson (D)	Pielou (Je)
WGF	0.62	0.36
MFC	0.81	0.61
MFO	0.89	0.71
PFA	0.94	0.83
PFR	0.92	0.77
PAU	0.73	0.56
PAA	0.93	0.81
PAR1	0.87	0.64
PAR2	0.75	0.51

*The Simpson (D) and Pielou (Je) indexes were calculated for all samples to measure their diversity and evenness, respectively. WGF, white guava fruits; MFC, closed medlar flowers; MFO, open medlar flowers; PFA, almost ripe passion fruits; PFR, ripe passion fruits; PAU, unripe papaya fruits; PAA, almost ripe papaya fruits; PAR1 and PAR2, ripe papaya fruits.*

## Discussion

In the current exploratory study, the composition of the epiphytic microbiomes present on the surfaces of different tropical fruits sampled in the Northern part of Argentina, known for its specific hot and humid climate, was investigated using shotgun metagenomics. Shotgun metagenomics has not been applied to analyze the surface microbiome of freshly picked fruits before, so this study can lay the basis for further research. A fraction of the metagenomic sequence reads could not be identified, notwithstanding the fact that a combination of different software tools and databases was used to obtain a good view on the microbial community diversity of complex ecosystems (Illeghems et al., 2012; Verce et al., 2019). Although Ion Torrent sequencing is known to have a homopolymer problem, the sequencing coverage was sufficient and this sequencing technique has shown to be reliable in food fermentation processes (Verce et al., 2019; De Roos et al., 2020). Taking this combined approach into account, a large variability in the epiphytic microbiome composition, both at genus and species level, could be unraveled among the different fruits (exemplified by white guava, passion fruit, and papaya, each of which were collected during one harvest), between the different ripening stages of a given fruit type (passion fruit and papaya) as well as between samples of a specific fruit type at the same ripening stage (papaya).

The species diversity of epiphytic microbiomes is known to vary among plant species. For instance, polymerase chain reaction-denaturing gradient gel electrophoresis (PCR-DGGE) has shown that the bacterial communities of the leaf phyllosphere of citrus trees, cotton, sugar beet, corn, and green bean can be distinct (Yang et al., 2001). This variation has also been found within plant species over the growing season, with in general the lowest species diversity being present just after emergence (Lindow and Brandl, 2003; Whipps et al., 2008). Furthermore, this species diversity is subjected to environmental events that influence the migration of microorganisms to the phyllosphere (Lindow and Brandl, 2003; Whipps et al., 2008; Berg et al., 2016). Indeed, during flowering, morphological changes in stigmata and nectaries form new niches for microbial colonization (Shade et al., 2013). Also, modification of the nutritional status of the surface of the fruits during ripening can attract a larger and/or more diverse microbial population, as has been shown for the surface of mango fruits at different harvesting stages (Jha et al., 2010; Gapper et al., 2013). However, this tendency could not be confirmed with the fruit samples of the current study. The species diversity of the almost ripe papaya and passion fruits was higher than that of the corresponding ripe ones. Also, the Japanese medlar flowers, which come first in the flower-fruit axis, had an overall higher species diversity than the fruits. A plausible reason for this tendency could be the fact that the present study applied a culture-independent, shotgun metagenomics approach, whereas previous studies have used culture-dependent techniques that target specific groups of microorganisms. Those techniques introduce a culturing bias, because the cultivation media used are not capable of capturing the full species diversity (Raspor et al., 2006; Chavan et al., 2009; Di Cagno et al., 2010, 2011a,^2013^; Li et al., 2010; Pelliccia et al., 2011; Pozo et al., 2012; Vadkertiová et al., 2012; Pontonio et al., 2018; Ruiz Rodríguez et al., 2019; Crespo et al., 2021; Gaglio et al., 2021).

Although a taxonomic identification approach was used to process the metagenomic sequence reads of the present study, which relied on four different classification methods in combination with fragment recruitment plotting, a large part of the MSRs could not be identified at species level for most samples. Nevertheless, the FRP method used has proven its merits in delivering a good view on species level identification of microorganisms from food fermentation microbiomes, which can have a very specific composition, as has been shown before for water kefir (Verce et al., 2019) and lambic beer (De Roos et al., 2020). Taking into account that the database to which the MSRs were mapped contained a representative genome sequence of each species of all genera identified in the samples investigated, extended with genome sequences of LAB and AAB species, this high number of non-identified MSRs could only be explained by the fact that species present in this specific niche were underrepresented in the NCBI assembly database and/or that novel species were present in the epiphytic microbiomes of the fruits examined (Yang et al., 2001; Shade et al., 2013). Underrepresentation of species in public sequence databases has also been reported for the apple carposphere (Angeli et al., 2019), exemplified by the presence of *Microbacterium* in fruit and flower samples of the present study, for which identification down to the species level using FRPs was not possible. Indeed, from the 117 species known for the genus *Microbacterium* at the time of analysis, only 61 species had a genome sequence available in the NCBI Assembly database. Due to this information gap, it is not clear whether already existing, non-sequenced species occurred, or that novel, non-characterized ones were present.

The microbial species present on the fruit and flower surfaces examined represented Proteobacteria (with a majority of enterobacteria and including AAB), Actinobacteria, Firmicutes (including LAB), and fungi (both molds and yeasts). Whereas LAB, AAB, and yeasts play a functional role in food fermentation processes, enterobacteria and several other aerobic and anaerobic bacteria can be phytopathogenic (Leroy and De Vuyst, 2004; Leff and Fierer, 2013). Genera known for colonizing plants, resulting in beneficial plant-microbiota interactions, characterized the surfaces of the fruits examined during the present study, including *Methylobacterium* and *Sphingomonas*, representing the bacterial core microbial community of apples (Delmotte et al., 2009; Vorholt, 2012; Rastogi et al., 2013; Shade et al., 2013; Vandenkoornhuyse et al., 2015; Liu et al., 2018; Abdelfattah et al., 2021). Whereas *Methylobacterium* was only identified at genus level during the present study, without the possibility to obtain species-level information because of the lack of sequenced genomes, several species of *Sphingomonas* occurred in different samples. Besides beneficial microorganisms, fruit surfaces also harbor phytopathogens (*Alternaria*, *Colletotrichum*, and *Erwinia* in the present study) that can be responsible for significant economic losses, before or after the harvest, although the indigenous microbiota present can determine and affect the outcome of plant-pathogen interactions and eventual infections (Vadkertiová et al., 2012; Abdelfattah et al., 2016). In the selected fruit units of the current study, no defects caused by phytopathogens were detected, although they can still be present on the fruits post-harvest. For papaya, anthracnose is a widespread post-harvest disease that lowers its commercial value and is caused by *Colletotrichum gloeosporioides* (Shi et al., 2010). However, *Colletotrichum* could not be identified at species level in the case of the papaya samples. *Alternaria* is another genus known to represent fungal pathogens, for instance responsible for core rot in apples (Abdelfattah et al., 2016), and it is representative for the surfaces of several fruits, *in casu* passion fruit and papaya. Also *Erwinia*, in particular *Erwinia amylovora*, is a phytopathogenic bacterium, causing fire blight on apples and pears (Lindow and Brandl, 2003; Shade et al., 2013; Aleklett et al., 2014; Junker and Keller, 2015). Nevertheless, this species nor other *Erwinia* species were identified on the fruit surfaces during the current study; however, one or more species related to *Erwinia dacicola* and *Erwinia iniecta* were present on the medlar flowers.

Typical food-borne pathogens, such as *Escherichia coli*, *Salmonella enterica*, *Shigella dysenteriae*, and *Staphylococcus aureus* were not present, assumably due to the sterile sampling of the fruits during the current study, while they usually contaminate fruits because of post-harvest handling (Jackson et al., 2015). Yet, other species of *Staphylococcus* were detected. Indeed, *Staphylococcus* has been identified as one of the core microbial genera of Firmicutes on oregano plants and also occurs as endophytic genus on papaya (Krishnan et al., 2012; Pontonio et al., 2018).

Some Gram-negative bacterial species of a certain genus are beneficial or pathogenic. *Stenotrophomonas* spp. are seen as potential plant-beneficial bacteria, but some species, such as *Stenotrophomonas maltophilia* are opportunistic pathogens for both plants and humans (Köberl et al., 2015). *Stenotrophomonas rhizophila*, however, is a model bacterium for plant growth promotors and stress-protecting agents (Köberl et al., 2015; Manirajan et al., 2016). As several *Pseudomonas* species are known colonizers of plants, some can be beneficial for the plant and others can be opportunistic pathogens for plants and/or humans (Delmotte et al., 2009; Shi et al., 2010; Vorholt, 2012; Rastogi et al., 2013; Köberl et al., 2015; Kim et al., 2018; Yu et al., 2019). *Pseudomonas putida* has been reported as antagonist of soil-borne pathogens, such as *Verticillium dahliae*, and has a role in suppressing the apple replant disease (Kim et al., 2018). Another known phytopathogenic bacterial genus is *Xanthomonas* (Kim et al., 2012; Vorholt, 2012; Liu et al., 2018; Yu et al., 2019). *Pantoea* spp. are often present at the highest relative abundances; however, some species of this genus are plant pathogens, whereas others can help protect their host from disease or even promote its growth (Vorholt, 2012; Leff and Fierer, 2013). *Enterobacter* can be identified on almost all apple tissues, when using high-throughput partial 16S RNA gene sequencing, and has also been recognized as a consistent and very abundant microbial community member of the plant phyllosphere using traditional culturing techniques (Leff and Fierer, 2013; Jackson et al., 2015; Wassermann et al., 2019). The most abundant species on the white guava sample, *Lelliottia nimipressularis*, has been isolated from water sources, food products, and tropical air and is known to be involved in the wetwood disease of trees (Heinle et al., 2018).

Whereas some species of the food fermentation-related microbial groups may have been overlooked before, given the microbiological analysis methods used, food-related ones may be used as autochthonous starter cultures in future fruit fermentation processes (Di Cagno et al., 2013; Ruiz Rodríguez et al., 2019; Crespo et al., 2021; Gaglio et al., 2021). Concerning LAB, *Lactococcus lactis*, *Leuconostoc citreum*, a species related to *Leuconostoc pseudomesenteroides*, and *Weissella cibaria* were present on the fruits and flowers examined, pointing to their omnipresence in such niches investigated, although all species witnessed very low relative abundances. Applying a culture-dependent approach, LAB species (in particular *L. pseudomesenteroides*, *Leuconostoc mesenteroides*, *Weissella minor*, *Levilactobacillus brevis*, and *Lactiplantibacillus plantarum*) have been isolated from the surfaces and/or pulps of several fruits (guava, passion fruit, and papaya) and medlar flowers from Northern Argentina before (Ruiz Rodríguez et al., 2019). Yet, differences occurred between the former culture-dependent analysis and the shotgun metagenomic methodology of the present study in that the more abundant species could be identified with both techniques, in contrast with the lesser abundant species, likely due to the methodologies applied, as the culture-dependent method relied on enrichment. For instance, shotgun metagenomics identified *W. cibaria* and a species related to *Weissella bombi* on open medlar flowers, whereas the species occurring culture-dependently were only found on the open flowers and not on the closed ones with this technique. These findings were in line with other studies applying culture-dependent identification methods targeting LAB associated with fresh fruits and spontaneously fermented fruits (Di Cagno et al., 2013; Yu et al., 2019; Crespo et al., 2021; Gaglio et al., 2021). Species of *Enterococcus*, more precisely *E. casseliflavus* (several fruits) and *Enterococcus faecium* (closed medlar flowers), were present as well. These LAB species have been isolated from plants before, but are not always desirable to use in food fermentation processes, as some species of this genus can even be pathogenic for humans, whereas others contribute to the final fermented food flavor (Foulquié Moreno et al., 2006; Ottesen et al., 2009). Fructophilic LAB that are often present in fructose-rich niches, such as flowers and fruits (Endo et al., 2009; Endo, 2012), were not represented, although genome sequences of fructophilic LAB were added on purpose to the in-house database used to construct the FRPs. This is in line with the results of the culture-dependent study of Ruiz Rodríguez et al. (2019) that has determined fructophilic LAB on custard apple, fig, and khaki, but not on the fruit types targeted in the current study.

Concerning AAB, two species of *Gluconobacter*, namely *Gluconobacter frateurii* and *Gluconobacter lacunae*, occurred on white guava. *Gluconobacter* has been characterized before on the carposphere of grapes using culture-independent techniques (Kecskeméti et al., 2016) and is also known to be a common nectar-inhabiting bacterium that may decrease the nectar attractiveness by altering the sugar composition (Aleklett et al., 2014). Besides LAB, AAB that are present in (fermented) fruit ecosystems may be used as starter cultures for food fermentation processes (Lisdiyanti et al., 2003; Pothakos et al., 2016; De Roos and De Vuyst, 2018).

Regarding yeasts, *Hanseniaspora* and *Metschnikowia* are common inhabitants of flowers and fruits. *Hanseniaspora* species have been encountered in alcoholization during fruit fermentation before (Bokulich et al., 2013). For instance, culture-dependent analysis of ripe grape berries has shown that *Hanseniaspora* is the predominant fungal genus, besides *Candida*, *Cryptococcus*, *Rhodotorula*, *Pichia*, *Metschnikowia* and/or *Kluyveromyces* (Raspor et al., 2006). Also, *Hanseniaspora* species are considered as important members of the cocoa fermentation microbiota, as they are retrieved from many spontaneous cocoa fermentation processes, independent of their origin (Ivory Coast, Brazil, Costa Rica, Ecuador, and Malaysia) or fermentation method applied (heap, box, or platform; Papalexandratou and De Vuyst, 2011; Díaz-Muñoz and De Vuyst, 2021). The presence of the *Metschnikowia* clade on flowers has been shown before, as its members have been encountered in nectar and pollinator communities, such as bumblebees (Aleklett et al., 2014). *Metschnikowia pulcherrima* is a known nectar- and fruit-inhabiting species, transferred by insects, and has been isolated from apple buds and flowers; it is further known to protect crops and agricultural products against pathogenic microorganisms (Pelliccia et al., 2011; Vadkertiová et al., 2012; Sipiczki, 2020).

To conclude, large differences in microbial community composition occurred between the different tropical fruit samples examined, both between and within the different fruit and flower types. The microbial fruit and flower communities unraveled contained mainly bacteria, as most metagenomic sequence reads corresponded with bacteria, whereas only a small number corresponded with yeasts. The majority of the genera and/or species identified were known inhabitants of flowers, fruits, and/or the phyllosphere. The fact that not all species that were part of these communities could be identified indicated that possibly some novel still to be explored species were present, or that representative genome sequences of those species were not yet available. Indeed, the underrepresentation or even the lack of microbial genomes related to environmental genera in public databases is so far still a drawback for studies as the current one. Species of LAB, AAB, and yeasts, suitable for usage in food fermentation processes, were only minor members within the microbial communities investigated, and no additional species occurred compared to previous culture-dependent studies focusing on fruit and fruit fermentation ecosystems. Based on the results of the present study, a culture-dependent selection strategy can be developed to define possible autochthonous fruit fermentation starter cultures. The analysis of the surface microbiota of the different fruits sampled in Northern Argentina can also help in tackling post-harvest diseases causing food losses between the harvest and consumption of the fruits.

## Data Availability Statement

The datasets presented in this study can be found in online repositories. The names of the repository/repositories and accession number(s) can be found below: https://www.ebi.ac.uk/ena, PRJEB50285.

## Author Contributions

LV and MV performed the sampling, the metagenomic DNA extraction, and sequencing. LV performed the bioinformatic analyses. FM and LDV contributed to the coordination of the sampling. LDV and SW designed and supervised the work and were responsible for funding. LV drafted the manuscript. LV, LDV, and SW revised the manuscript. All authors read and approved the final version of the manuscript.

## Conflict of Interest

The authors declare that the research was conducted in the absence of any commercial or financial relationships that could be construed as a potential conflict of interest.

## Publisher’s Note

All claims expressed in this article are solely those of the authors and do not necessarily represent those of their affiliated organizations, or those of the publisher, the editors and the reviewers. Any product that may be evaluated in this article, or claim that may be made by its manufacturer, is not guaranteed or endorsed by the publisher.

## References

[B1] Abdelfattah A. Freilich S. Bartuv R. Zhimo V. Y. Kumar A. Biasi A. (2021). Global analysis of the apple fruit microbiome: are all apples the same? *Environ. Microbiol.* 23 6038–6055. 10.1111/1462-2920.15469 33734550 PMC8596679

[B2] Abdelfattah A. Wisniewski M. Droby S. Schena L. (2016). Spatial and compositional variation in the fungal communities of organic and conventionally grown apple fruit at the consumer point-of-purchase. *Hortic. Res.* 3:16047. 10.1038/hortres.2016.47 27766161 PMC5051542

[B3] Ailes E. C. Leon J. S. Jaykus L.-A. Johnston L. M. Clayton H. A. Blanding S. (2008). Microbial concentrations on fresh produce are affected by postharvest processing, importation, and season. *J. Food Prot.* 71 2389–2397. 10.4315/0362-028X-71.12.2389 19244889

[B4] Aleklett K. Hart M. Shade A. (2014). The microbial ecology of flowers: an emerging frontier in phyllosphere research. *Botany* 92 253–266. 10.1139/cjb-2013-0166

[B5] Altschul S. F. Gish W. Miller W. Myers E. W. Lipman D. J. (1990). Basic local alignment search tool. *J. Mol. Biol.* 215 403–410. 10.1016/S0022-2836(05)80360-2 2231712

[B6] Anderson M. J. (2017). Permutational multivariate analysis of variance (PERMANOVA). *Wiley StatsRef: Statistics Reference Online*, 1–15. 10.1002/9781118445112.stat07841

[B7] Andrews S. (2010). *FastQC: A Quality Control Tool for High Throughput Sequence Data.* Available online at: http://www.bioinformatics.babraham.ac.uk/projects/fastqc (accessed January 6, 2022).

[B8] Angeli D. Sare A. R. Jijakli M. H. Pertot I. Massart S. (2019). Insights gained from metagenomic shotgun sequencing of apple fruit epiphytic microbiota. *Postharvest Biol. Technol.* 153 96–106. 10.1016/j.postharvbio.2019.03.020

[B9] Auguie B. (2017). *GridExtra: Miscellaneous Functions for “Grid” Graphics. R Package Version 2.3.* Available online at: http://CRAN.R-project.org/package=gridExtra (accessed January 6, 2022).

[B10] Batool F. Rehman Y. Hasnain S. (2016). Phylloplane associated plant bacteria of commercially superior wheat varieties exhibit superior plant growth promoting abilities. *Front. Life Sci.* 9:313–322. 10.1080/21553769.2016.1256842

[B11] Berg G. Rybakova D. Grube M. Köberl M. (2016). The plant microbiome explored: implications for experimental botany. *J. Exp. Bot.* 67 995–1002. 10.1093/jxb/erv466 26547794 PMC5395086

[B12] Bokulich N. A. Thorngate J. H. Richardson P. M. Mills D. A. (2013). Microbial biogeography of wine grapes is conditioned by cultivar, vintage, and climate. *Proc. Natl. Acad. Sci. U.S.A.* 11 E139–E148. 10.1073/pnas.1317377110 24277822 PMC3890796

[B13] Buchfink B. Xie C. Huson D. H. (2015). Fast and sensitive protein alignment using DIAMOND. *Nat. Methods* 12 59–60. 10.1038/nmeth.3176 25402007

[B14] Chavan P. Mane S. Kulkarni G. Shaikh S. Ghormade V. Nerkar D. P. (2009). Natural yeast flora of different varieties of grapes used for wine making in India. *Food Microbiol.* 26 801–808. 10.1016/j.fm.2009.05.005 19835764

[B15] Clarke K. R. (1993). Nonparametric multivariate analyses of changes in community structure. *Austral. Ecol.* 18 117–143. 10.1111/j.1442-9993.1993.tb00438.x

[B16] Core Team R. (2020). *R: A Language and Environment for Statistical Computing.* Vienna: R Foundation for Statistical Computing.

[B17] Crespo L. Gaglio R. Martínez F. G. Martin G. M. Franciosi E. Madrid-Albarrrán Y. (2021). Bioaccumulation of selenium-by fruit origin lactic acid bacteria in tropical fermented fruit juices. *LWT* 151:112103. 10.1016/j.lwt.2021.112103

[B18] De Bruyn F. Zhang S. J. Pothakos V. Torres J. Lambot C. Moroni A. V. (2017). Exploring the impacts of postharvest processing on the microbiota and metabolite profiles during green coffee bean production. *Appl. Environ. Microbiol.* 83:e02398-16. 10.1128/AEM.02398-16 27793826 PMC5165123

[B19] De Roos J. De Vuyst L. (2018). Acetic acid bacteria in fermented foods and beverages. *Curr. Opin. Biotechnol.* 49 115–119. 10.1016/j.copbio.2017.08.007 28863341

[B20] De Roos J. Verce M. Weckx S. De Vuyst L. (2020). Temporal shotgun metagenomics revealed the potential metabolic capabilities of specific microorganisms during lambic beer production. *Front. Microbiol.* 11:1692. 10.3389/fmicb.2020.01692 32765478 PMC7380088

[B21] Delmotte N. Knief C. Chaffron S. Innerebner G. Roschitzki B. Schlapbach R. (2009). Community proteogenomics reveals insights into the physiology of phyllosphere bacteria. *Proc. Natl. Acad. Sci. U.S.A.* 106 16428–16433. 10.1073/pnas.0905240106 19805315 PMC2738620

[B22] Di Cagno R. Cardinali G. Minervini G. Antonielli L. Rizzello C. G. Ricciuti P. (2010). Taxonomic structure of the yeasts and lactic acid bacteria microbiota of pineapple (*Ananas comosus* L. Merr.) and use of autochthonous starters for minimally processing. *Food Microbiol.* 27 381–389. 10.1016/j.fm.2009.11.012 20227603

[B23] Di Cagno R. Minervini G. Rizzello C. G. De Angelis M. Gobbetti M. (2011a). Effect of lactic acid fermentation on antioxidant, texture, color and sensory properties of red and green smoothies. *Food Microbiol.* 28 1062–1071. 10.1016/j.fm.2011.02.011 21569953

[B24] Di Cagno R. Surico R. F. Minervini G. Rizzello C. G. Lovino R. Servili M. (2011b). Exploitation of sweet cherry (*Prunus avium* L.) puree added of stem infusion through fermentation by selected autochthonous lactic acid bacteria. *Food Microbiol.* 28 900–909. 10.1016/j.fm.2010.12.008 21569932

[B25] Di Cagno R. Coda R. De Angelis M. Gobbetti M. (2013). Exploitation of vegetables and fruits through lactic acid fermentation. *Food Microbiol.* 33 1–10. 10.1016/j.fm.2012.09.003 23122495

[B26] Di Cagno R. Filannino P. Gobbetti M. (2016a). “Novel fermented fruit and vegetable-based products,” in *Novel Food Fermentation Technologies, Food Engineering Series*, eds Ojha K. S. Tiwari B. K. (Cham: Springer), 279–291.

[B27] Di Cagno R. Filannino P. Vincentini O. Lanera A. Cavoski I. Gobbetti M. (2016b). Exploitation of *Leuconostoc mesenteroides* strains to improve shelf life, rheological, sensory and functional features of prickly pear (*Opuntia ficus-indica* L.) fruit puree. *Food Microbiol.* 59 176–189. 10.1016/j.fm.2016.06.009 27375258

[B28] Di Cagno R. Filannino P. Cavoski I. Lanera A. Mamdouh B. M. Gobbetti M. (2017). Bioprocessing technology to exploit organic palm date (*Phoenix dactylifera* L. cultivar Siwi) fruit as a functional dietary supplement. *J. Funct. Foods* 31 9–19. 10.1016/j.jff.2017.01.033

[B29] Díaz-Muñoz C. De Vuyst L. (2021). Functional starter cultures for cocoa fermentation. *J. Appl. Microbiol.* 1–28. 10.1111/jam.15312 34599633 PMC9542016

[B30] Endo A. (2012). Fructophilic lactic acid bacteria inhabit fructose-rich niches in nature. *Microb. Ecol. Health Dis.* 23:18563. 10.3402/mehd.v23i0.18563 23990834 PMC3747758

[B31] Endo A. Futagawa-Endo Y. Dicks L. M. T. (2009). Isolation and characterization of fructophilic lactic acid bacteria from fructose-rich niches. *Syst. Appl. Microbiol.* 32 593–600. 10.1016/j.syapm.2009.08.002 19733991

[B32] Foulquié Moreno M. R. Sarantinopoulos P. Tsakalidou E. De Vuyst L. (2006). The role and application of enterococci in food and health. *Int. J. Food Microbiol.* 106 1–24. 10.1016/j.ijfoodmicro.2005.06.026 16216368

[B33] Gaglio R. Pescuma M. Madrid-Albarrán Y. Franciosi E. Moschetti G. Francesca N. (2021). Selenium bio-enrichment of Mediterranean fruit juices through lactic acid fermentation. *Int. J. Food Microbiol.* 354:109248. 10.1016/j.ijfoodmicro.2021.109248 34059319

[B34] Gapper N. E. McQuinn R. P. Giovannoni J. J. (2013). Molecular and genetic regulation of fruit ripening. *Plant Mol. Biol.* 82 575–591. 10.1007/s11103-013-0050-3 23585213

[B35] Geyzen A. Scholliers P. Leroy F. (2012). Innovative traditions in swiftly transforming foodscapes: an exploratory essay. *Trends Food Sci. Technol.* 25 47–52. 10.1016/j.tifs.2011.12.003

[B36] Heaton J. C. Jones K. (2008). Microbial contamination of fruit and vegetables and the behaviour of enteropathogens in the phyllosphere: a review. *J. Appl. Microbiol.* 104 613–626. 10.1111/j.1365-2672.2007.03587.x 17927745

[B37] Heinle C. E. Junqueira A. C. M. Uchida A. Purbojati R. W. Houghton J. N. I. Chénard C. (2018). Complete genome sequence of *Lelliottia nimipressularis* type strain SGAir0187, isolated from tropical air collected in Singapore. *Genome Announc.* 6:e00231-18. 10.1128/genomeA.00231-18 29724826 PMC5940956

[B38] Hervé M. (2021). *RVAideMemoire: Testing and Plotting Procedures for Biostatistics. R package version 0.9.79.* Available online at: https://CRAN.R-project.org/package=RVAideMemoire (accessed January 6, 2022).

[B39] Hugenholtz J. (2013). Traditional biotechnology for new foods and beverages. *Curr. Opin. Biotechnol.* 24 155–159. 10.1016/j.copbio.2013.01.001 23395405

[B40] Huson D. Auch A. Qi J. Schuster S. (2007). MEGAN analysis of metagenome data. *Genome Res.* 17 377–386. 10.1101/gr.5969107 17255551 PMC1800929

[B41] Hutkins R. W. (2019). “Fermented vegetables,” in *Microbiology and Technology of Fermented Foods*, ed. Hutkins R. W. (Oxford: Blackwell Publishing), 267–300.

[B42] Illeghems K. De Vuyst L. Papalexandratou Z. Weckx S. (2012). Phylogenetic analysis of a spontaneous cocoa bean fermentation metagenome reveals new insights into its bacterial and fungal community diversity. *PLoS One* 7:e38040. 10.1371/journal.pone.0038040 22666442 PMC3362557

[B43] Isas A. S. Mariotti Celis M. S. Pérez Correa J. R. Fuentes E. Rodríguez L. Palomo I. (2020). Functional fermented cherimoya (*Annona cherimola* Mill.) juice using autochthonous lactic acid bacteria. *Food Res. Int.* 138:109729. 10.1016/j.foodres.2020.109729 33292965

[B44] Jackson C. R. Stone B. W. G. Tyler H. L. (2015). Emerging perspectives on the natural microbiome of fresh produce vegetables. *Agriculture* 5 170–187. 10.3390/agriculture5020170

[B45] Jha S. N. Jaiswal P. Narsaiah K. Bhardwaj R. Sharma R. Kumar R. (2010). Post-harvest micro-flora on major cultivars of Indian mangoes. *Sci. Hortic.* 125 617–621. 10.1016/j.scienta.2010.05.011

[B46] Junker R. R. Keller A. (2015). Microhabitat heterogeneity across leaves and flower organs promotes bacterial diversity. *FEMS Microbiol. Ecol.* 91:fiv097. 10.1093/femsec/fiv097 26253507

[B47] Kecskeméti E. Berkelmann-Löhnertz B. Reineke A. (2016). Are epiphytic microbial communities in the carposphere of ripening grape clusters (*Vitis vinifera* L.) different between conventional, organic, and biodynamic grapes? *PLoS One* 11:e0160852. 10.1371/journal.pone.0160852 27500633 PMC4976965

[B48] Kim M. Singh D. Lai-Hoe A. Chun J. Adams J. M. (2012). Distinctive phyllosphere bacterial communities in tropical trees. *Microb. Ecol.* 63 674–681. 10.1007/s00248-011-9953-1 21990015

[B49] Kim M.-J. Jeon C.-W. Cho G. Kim D.-R. Kwack Y.-B. Kwak Y.-S. (2018). Comparison of microbial community structure in kiwifruit pollens. *Plant Pathol. J.* 34 143–149. 10.5423/PPJ.NT.12.2017.0281 29628821 PMC5880359

[B50] Köberl M. Dita M. Martinuz A. Staver C. Berg G. (2015). Agroforestry leads to shifts within the gammaproteobacterial microbiome of banana plants cultivated in central America. *Front. Microbiol.* 6:91. 10.3389/fmicb.2015.00091 25717322 PMC4324142

[B51] Krishnan P. Bhat R. Kush A. Ravikumar P. (2012). Isolation and functional characterization of bacterial endophytes from *Carica papaya* fruits. *J. Appl. Microbiol.* 113 308–317. 10.1111/j.1365-2672.2012.05340.x 22587617

[B52] Kusstatscher P. Cernava T. Abdelfattah A. Gokul J. Korsten L. Berg G. (2020). Microbiome approaches provide the key to biologically control postharvest pathogens and storability of fruits and vegetables. *FEMS Microbiol. Ecol.* 96:fiaa119. 10.1093/femsec/fiaa119 32542314

[B53] Leff J. W. Fierer N. (2013). Bacterial communities associated with the surfaces of fresh fruits and vegetables. *PLoS One* 8:e59310. 10.1371/journal.pone.0059310 23544058 PMC3609859

[B54] Leroy F. De Vuyst L. (2004). Lactic acid bacteria as functional starter cultures for the food fermentation industry. *Trends Food Sci. Technol.* 15 67–78. 10.1016/j.tifs.2003.09.004

[B55] Li S.-S. Cheng C. Li Z. Chen J.-Y. Yan B. Han B.-Z. (2010). Yeast species associated with wine grapes in China. *Int. J. Food Microbiol.* 138 85–90. 10.1016/j.ijfoodmicro.2010.01.009 20116124

[B56] Lindow S. E. Brandl M. T. (2003). Microbiology of the phyllosphere. *Appl. Environ. Microbiol.* 69 1875–1883. 10.1128/AEM.69.4.1875-1883.2003 12676659 PMC154815

[B57] Lisdiyanti P. Katsura K. Potacharoen W. Navarro R. R. Yamada Y. Uchimura T. (2003). Diversity of acetic acid bacteria in Indonesia, Thailand, and the Philippines. *Microbiol. Cult. Collect.* 19 91–99.

[B58] Liu J. Abdelfattah A. Norelli J. Burchard E. Schena L. Droby S. (2018). Apple endophytic microbiota of different rootstock/scion combinations suggests a genotype-specific influence. *Microbiome* 6:18. 10.1186/s40168-018-0403-x 29374490 PMC5787276

[B59] Manirajan B. A. Ratering S. Rusch V. Schwiertz A. Geissler-Plaum R. Cardinale M. (2016). Bacterial microbiota associated with flower pollen is influenced by pollination type, and shows a high degree of diversity and species-specificity. *Environ. Microbiol.* 18 5161–5174. 10.1111/1462-2920.13524 27612299

[B60] Marco M. L. Heeney D. Binda S. Cifelli C. J. Cotter P. D. Foligné B. (2017). Health benefits of fermented foods: microbiota and beyond. *Curr. Opin. Biotechnol.* 44 94–102. 10.1016/j.copbio.2016.11.010 27998788

[B61] Marco M. L. Sanders M. E. Gänzle M. Arrieta M. C. Cotter P. D. De Vuyst L. (2021). The international scientific association for probiotics and prebiotics (ISAPP) consensus statement on fermented foods. *Nat. Rev. Gastroenterol. Hepatol.* 18 196–208. 10.1038/s41575-020-00390-5 33398112 PMC7925329

[B62] Menzel P. Ng K. L. Krogh A. (2016). Fast and sensitive taxonomic classification for metagenomics with Kaiju. *Nat. Commun.* 7:11257. 10.1038/ncomms11257 27071849 PMC4833860

[B63] Mouillot D. Leprêtre A. (1999). A comparison of species diversity estimators. *Res. Popul. Ecol.* 41 203–215. 10.1007/s101440050024

[B64] Oksanen J. Blanchet F. G. Friendly M. Kindt R. Legendre P. McGlinn D. (2019). *Vegan Community Ecology Package. R Package Version 2.5-4.* Available online at: https://CRAN.R-project.org/package=vegan (accessed January 6, 2022).

[B65] Ottesen A. R. González Peña A. White J. R. Pettengill J. B. Li C. Allard S. (2013). Baseline survey of the anatomical microbial ecology of an important food plant: *Solanum lycopersicum* (tomato). *BMC Microbiol.* 13:114. 10.1186/1471-2180-13-114 23705801 PMC3680157

[B66] Ottesen A. R. White J. R. Skaltsas D. N. Newell M. J. Walsh C. S. (2009). Impact of organic and conventional management on the phyllosphere microbial ecology of an apple crop. *J. Food Prot.* 72 2321–2325. 10.4315/0362-028X-72.11.2321 19903395

[B67] Papalexandratou Z. De Vuyst L. (2011). Assessment of the yeast species composition of cocoa bean fermentations in different cocoa-producing regions using denaturating gradient gel electrophoresis. *FEMS Yeast Res.* 11 564–574. 10.1111/j.1567-1364.2011.00747.x 22093683

[B68] Pelliccia C. Antonielli L. Corte L. Bagnetti A. Fatichenti F. Cardinali G. (2011). Preliminary prospection of the yeast biodiversity on apple and pear surfaces from Northern Italy orchards. *Ann. Microbiol.* 61 965–972. 10.1007/s13213-011-0220-y

[B69] Pontonio E. Di Cagno R. Tarraf W. Filannino P. De Mastro G. Gobbetti M. (2018). Dynamic and assembly of epiphyte and endophyte lactic acid bacteria during the life cycle of *Origanum vulgare* L. *Front. Microbiol.* 9:1372. 10.3389/fmicb.2018.01372 29997592 PMC6029521

[B70] Pothakos V. Illeghems K. Laureys D. Spitaels F. Vandamme P. De Vuyst L. (2016). “Acetic acid bacteria in fermented food and beverage ecosystems,” in *Acetic Acid Bacteria*, Ecology and Physiology, eds Matsushita K. Toyama H. Tonouchi N. Okamoto-Kainuma A. (Tokyo: Springer), 73–100.

[B71] Pozo M. I. Lachance M.-A. Herrera C. M. (2012). Nectar yeasts of two southern Spanish plants: the roles of immigration and physiological traits in community assembly. *FEMS Microbiol. Ecol.* 80 281–293. 10.1111/j.1574-6941.2011.01286.x 22224447

[B72] Randazzo W. Corona O. Guarcello R. Francesca N. Germanà M. A. Erten H. (2016). Development of new non-dairy beverages from Mediterranean fruit juices fermented with water kefir microorganisms. *Food Microbiol.* 54 40–51. 10.1016/j.fm.2015.10.018

[B73] Raspor P. Milek D. M. Polanc J. Smole Možina S. Čadež N. (2006). Yeasts isolated from three varieties of grapes cultivated in different locations of the Dolenjska vine-growing region, Slovenia. *Int. J. Food Microbiol.* 109 97–102. 10.1016/j.ijfoodmicro.2006.01.017 16626833

[B74] Rastogi G. Coaker G. L. Leveau J. H. J. (2013). New insights into the structure and function of phyllosphere microbiota through high-throughput molecular approaches. *FEMS Microbiol. Lett.* 348 1–10. 10.1111/1574-6968.12225 23895412

[B75] Redford A. J. Bowers R. M. Knight R. Linhart Y. Fierer N. (2010). The ecology of the phyllosphere: geographic and phylogenetic variability in the distribution of bacteria on tree leaves. *Environ. Microbiol.* 12 2885–2893. 10.1111/j.1462-2920.2010.02258.x 20545741 PMC3156554

[B76] RStudio Team. (2020). *RStudio: Integrated Development for R.* Boston, MA: RStudio.

[B77] Ruiz Rodríguez L. G. Mohamed F. Bleckwedel J. Medina R. De Vuyst L. Hebert E. M. (2019). Diversity and functional properties of lactic acid bacteria isolated from wild fruits and flowers present in Northern Argentina. *Front. Microbiol.* 10:1091. 10.3389/fmicb.2019.01091 31164879 PMC6536596

[B78] Schmieder R. Edwards R. (2011). Quality control and preprocessing of metagenomic datasets. *Bioinformatics* 27 863–864. 10.1093/bioinformatics/btr026 21278185 PMC3051327

[B79] Shade A. McManus P. S. Handelsman J. (2013). Unexpected diversity during community succession in the apple. *mBio* 4:e00602-12. 10.1128/mBio.00602-12 23443006 PMC3585449

[B80] Shi J. Liu A. Li X. Feng S. Chen W. (2010). Identification of endophytic bacterial strain MGP1 selected from papaya and its biocontrol effects on pathogens infecting harvested papaya fruit. *J. Sci. Food Agric.* 90 227–232. 10.1002/jsfa.3798 20355035

[B81] Sipiczki M. (2020). *Metschnikowia pulcherrima* and related pulcherrimin-producing yeasts: fuzzy species boundaries and complex antimicrobial antagonism. *Microorganisms* 8:1029. 10.3390/microorganisms8071029 32664630 PMC7409158

[B82] Slowikowski K. (2020). *ggrepel: Automatically Position Non-Overlapping Text Labels With ‘ggplot2’. R Package Version 0.8.2.* Available online at: https://CRAN.R-project.org/package=ggrepel (accessed January 6, 2022).

[B83] Vadkertiová R. Molnárová J. Vránová D. Sláviková E. (2012). Yeasts and yeast-like organisms associated with fruits and blossoms of different fruit trees. *Can. J. Microbiol.* 58 1344–1352. 10.1139/cjm-2012-0468 23210991

[B84] Vandenkoornhuyse P. Quaiser A. Duhamel M. Le Van A. Dufresne A. (2015). The importance of the microbiome of the plant holobiont. *New Phytol.* 206 1196–1206. 10.1111/nph.13312 25655016

[B85] Verce M. De Vuyst L. Weckx S. (2019). Shotgun metagenomics of a water kefir fermentation ecosystem reveals a novel *Oenococcus* species. *Front. Microbiol.* 10:479. 10.3389/fmicb.2019.00479 30918501 PMC6424877

[B86] Verce M. Schoonejans J. Hernandez Aguirre C. Molina-Bravo R. De Vuyst L. Weckx S. (2021). A combined metagenomics and metatranscriptomics approach to unravel Costa Rican cocoa box fermentation processes reveals yet unreported microbial species and functionalities. *Front. Microbiol.* 12:641185. 10.3389/fmicb.2021.641185 33664725 PMC7920976

[B87] Vermote L. Verce M. De Vuyst L. Weckx S. (2018). Amplicon and shotgun metagenomic sequencing indicates that microbial ecosystems present in cheese brines reflect environmental inoculation during the cheese production process. *Int. Dairy J.* 87 44–53. 10.1016/j.idairyj.2018.07.010

[B88] Vorholt J. A. (2012). Microbial life in the phyllosphere. *Nat. Rev. Microbiol.* 10 828–840. 10.1038/nrmicro2910 23154261

[B89] Wassermann B. Müller H. Berg G. (2019). An apple a day: which bacteria do we eat with organic and conventional apples? *Front. Microbiol.* 10:1629. 10.3389/fmicb.2019.01629 31396172 PMC6667679

[B90] Whipps J. M. Hand P. Pink D. Bending G. D. (2008). Phyllosphere microbiology with special reference to diversity and plant genotype. *J. Appl. Microbiol.* 105 1744–1755. 10.1111/j.1365-2672.2008.03906.x 19120625

[B91] Wickham H. (2007). Reshaping data with the reshape package. *J. Stat. Softw.* 21 1–20. 10.18637/jss.v021.i12

[B92] Wickham H. (2016). *ggplot2: Elegant Graphics for Data Analysis.* New York, NY: Springer-Verlag.

[B93] Wickham H. (2019). *Lazyeval: Lazy (Non-Standard) Evaluation. R package Version 0.2.2.* Available online at: https://CRAN.R-project.org/package=lazyeval (accessed January 6, 2022).

[B94] Wickham H. Seidel D. (2020). *Scales: Scale Functions for Visualization. R package version 1.1.1.* Available online at: https://CRAN.R-project.org/package=scales (accessed January 6, 2022).

[B95] Wickham H. Averick M. Bryan J. Chang W. McGowan L. D. François R. (2019). Welcome to the tidyverse. *J. Open Source Softw.* 4:1686. 10.21105/joss.01686

[B96] Wolfe B. E. Dutton R. J. (2015). Fermented foods as experimentally tractable microbial ecosystems. *Cell* 161 49–55. 10.1016/j.cell.2015.02.034 25815984

[B97] Wood D. E. Salzberg S. L. (2014). Kraken: ultrafast metagenomic sequence classification using exact alignments. *Genome Biol.* 15:R46. 10.1186/gb-2014-15-3-r46 24580807 PMC4053813

[B98] Yang C.-H. Crowley D. E. Borneman J. Keen N. T. (2001). Microbial phyllosphere populations are more complex than previously realized. *Proc. Natl. Acad. Sci. U.S.A.* 98 3889–3894. 10.1073/pnas.051633898 11274410 PMC31148

[B99] Yashiro E. Spear R. N. McManus P. S. (2011). Culture-dependent and culture-independent assessment of bacteria in the apple phyllosphere. *J. Appl. Microbiol.* 110 1284–1296. 10.1111/j.1365-2672.2011.04975.x 21332895

[B100] Yu A. O. Leveau J. H. J. Marco M. L. (2019). Abundance, diversity and plant-specific adaptations of plant-associated lactic acid bacteria. *Environ. Microbiol. Rep.* 12 16–29. 10.1111/1758-2229.12794 31573142

